# Retinal Detachment Accompanied by a Macular Hole in a Patient With ABCA4 and BEST1 Genetic Mutations

**DOI:** 10.7759/cureus.78477

**Published:** 2025-02-04

**Authors:** Basma Alqaseer, Mariam Bunajem

**Affiliations:** 1 Department of Ophthalmology, Salmaniya Medical Complex, Manama, BHR

**Keywords:** hereditary retinal diseases, inherited macular dystrophy, macular hole, retinal detachment, vitreoretinal surgery

## Abstract

Inherited macular dystrophies are a heterogeneous group of disorders characterized by loss of central vision due to macular and retinal pigment epithelium atrophy. Mutations include ABCA4 and BEST1 genes, which are found in different conditions such as Stargardt disease, Best disease, and also in age-related maculopathies. We report a case of retinal detachment and macular hole (MH) in a middle-aged patient with both ABCA4 and BEST1 mutations.

A 65-year-old man presented to the emergency room with a 10-day history of floaters, described as a gray curtain, in the left eye (LE). He denied a history of change in vision. The patient has a positive history of gene mutations in both ABCA4 and BEST1 genes, which was diagnosed a couple of years prior with a recorded best corrected visual acuity (BCVA) of 6/60 at that time. On presentation, the ophthalmic examination of the anterior segment was unremarkable in both eyes, with a BCVA of 6/9 and 6/60 in the right eye and LE, respectively. The affected eye showed retinal detachment with the macula off. Additionally, optical coherence tomography of the macula and B scan showed full-thickness MH with retinal detachment. He underwent pars plana vitrectomy with internal limiting membrane peeling. Postoperatively, the BCVA was 6/60.

Inherited retinal dystrophies may be associated with MH formation. Further studies are prudent to understand the pathophysiology of MHs and prevent subsequent complications such as retinal detachment, especially in patients with multiple gene variants.

## Introduction

Inherited macular dystrophies are a heterogeneous group of disorders characterized by loss of central vision associated with macular and retinal pigment epithelium (RPE) atrophy [1]. Mutations in ABCA4 and BEST1 genes were found in Stargardt disease (STGD) [2-4]. STGD is a common form of inherited macular dystrophy [3,4]. Most cases are inherited in an autosomal recessive pattern [3-5]. It is characterized by abnormal accumulation of lipofuscin in the RPE [4]. The most common presentation is central vision loss with macular atrophy and white flecks at the RPE level in the posterior pole [1].

Diagnosis is more commonly made in childhood. Late presentation of the disease may also occur but is less common and is associated with a better prognosis [5-7]. Histopathological changes in the vitreoretinal interface were reported in patients with STGD [4].

Age-related maculopathies have similar features seen in STGD [7]. The ABCA4 variants were also found in age-related macular degeneration (AMD) [5]. It is an acquired cause of central vision loss in the elderly [7]. Mutations in the BEST1 gene also cause Best disease, which is an autosomal dominant-inherited macular dystrophy [8]. It is clinically characterized by bilateral round yellow or egg-yolk macular lesions [1,8]. We report a rare presentation of retinal detachment and macular hole (MH) in a middle-aged patient with both ABCA4 and BEST1 mutations.

## Case presentation

**Figure 1:**
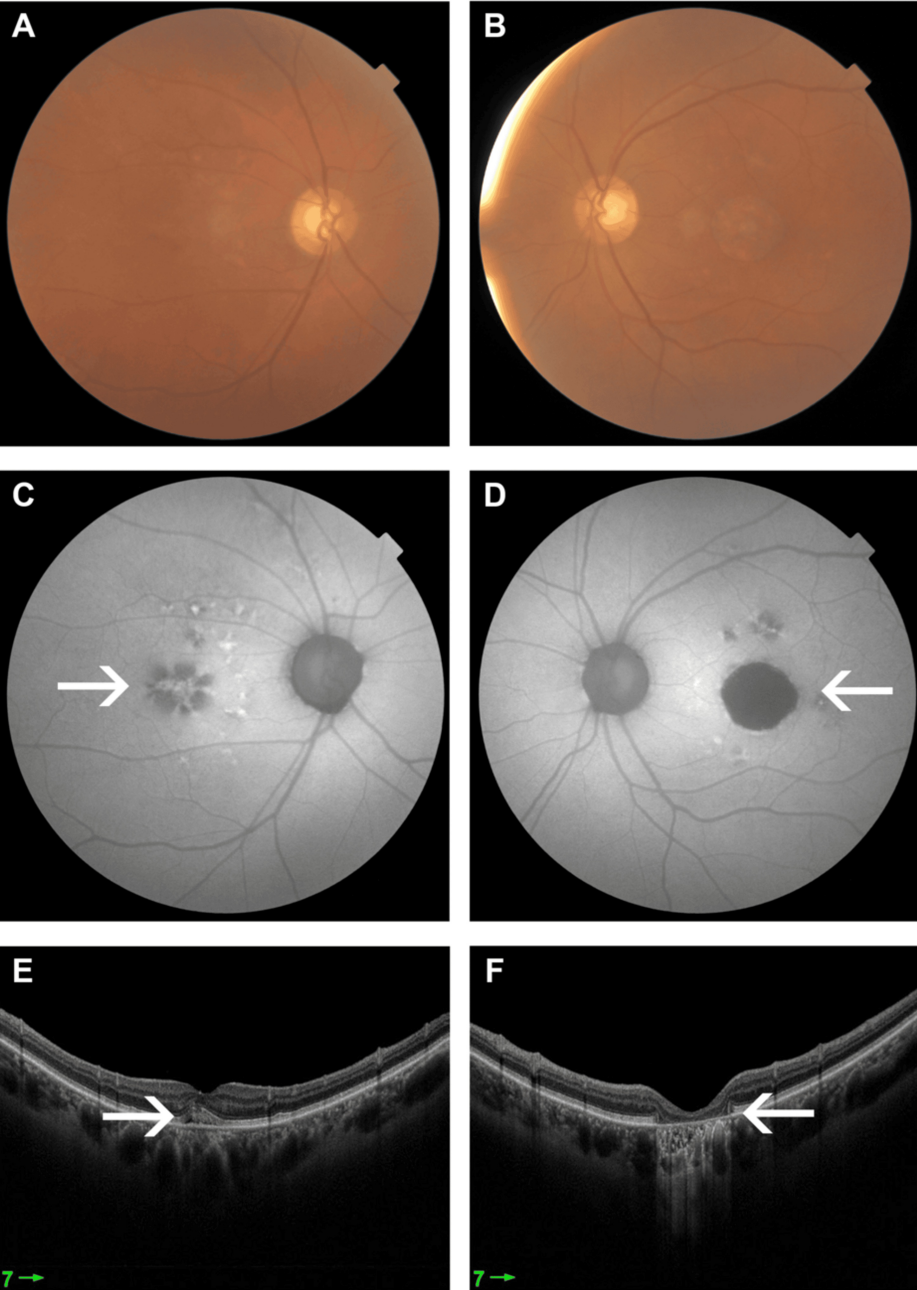
Colored fundus photograph of the RE (A) and LE (B) one year prior to presentation. Central hypoautofluorescence secondary to photoreceptor atrophy, surrounded by an area of hyperautofluorescence (white arrow) secondary to lipofuscin accumulation, more in the LE (D) compared to the RE (C). OCT showing photoreceptor loss more in the LE (F) than the RE (E) (white arrow) OCT: optical coherence tomography; LE: left eye; RE: right eye

A 65-year-old man presented to the emergency room with a 10-day history of floaters, described as a gray curtain, in the left eye (LE). He denied symptoms of change or a decrease in vision. He also denied a history of ocular trauma. The patient has a positive history of gene mutations in both ABCA4 and BEST1 genes, which was diagnosed a couple of years prior. His visual acuity on regular follow-up was stable and recorded as 6/60 over several years (Figure 1).

**Figure 2:**
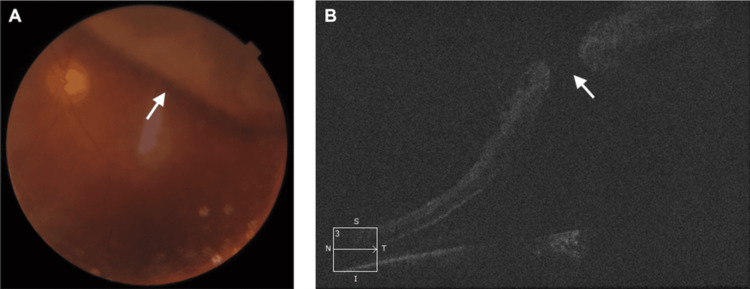
LE. (A) Colored fundus photograph showing retinal detachment (white arrow). (B) Raster OCT showing a full thickness MH with subretinal fluid (white arrow) OCT: optical coherence tomography; LE: left eye; MH: macular hole

On examination, the best corrected visual acuity (BCVA) was 6/9 in the right eye (RE) and 6/60 in the LE. Anterior segment examination was unremarkable in both eyes. A dilated fundus examination of the RE showed multiple submacular lesions with RPE changes, and the LE showed a superior bullous retinal detachment with the macula off. Further investigations with colored fundus photo and optical coherence tomography (OCT) of the macula showed full-thickness MH and retinal detachment in the LE (Figure 2).

**Figure 3:**
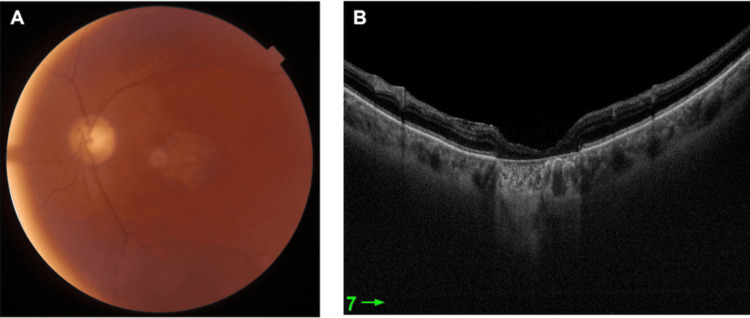
LE after silicone oil removal. (A) Colored fundus photograph. (B) OCT OCT: optical coherence tomography; LE: left eye

The patient was reviewed by the vitreoretinal service and subsequently underwent a 25-gauge triamcinolone-assisted pars plana vitrectomy. This procedure included internal limiting membrane peeling, endophotocoagulation laser treatment, air-fluid exchange, and silicone injection, all performed under local anesthesia in the LE. Intraoperatively, a superior retinal break and a full-thickness MH were noted. The patient was followed up postoperatively and BCVA was recorded as 6/60. Follow-up OCT showed complete closure of the MH (Figure 3).

## Discussion

Both ABCA1 and BEST1 gene mutations were identified in STGD [2-5]. Stargardt is a common dystrophy characterized by abnormal accumulation of lipofuscin in the RPE [4]. Lipofuscin accumulation in the RPE also occurs in AMD resulting in vision impairment [7]. Age-related maculopathies were found to have similar clinical features to STGD [7].

Although ABCA4 and BEST1 mutations are seen in STGD, they have also been reported in AMD and Best disease, respectively [5,8]. In the medical community, the consensus of this finding is variable, as some showed that ABCA4 alleles are elevated in AMD, while others showed the contrary [9]. It has been suggested that many AMD cases were misdiagnosed as late-onset ABCA4-related disease [9].

Vitreoretinal interface changes were associated with MH in young adults and children [4,10-13]. Two cases reported MH in young adults with STGD [4,13]. MH in a patient with both ABCA4 and BEST1 has never been reported in a middle-aged patient to our knowledge, as in our case.

Our patient underwent surgical intervention, achieving a good anatomical outcome and a BCVA of 6/60. A favorable surgical outcome was reported previously in a patient with STGD [4].​​​​​​​

It was hypothesized that in STGD, RPE changes cause a reduced pumping effect, which results in poor adherence of RPE to the neurosensory retina. The macular area is associated with both RPE and photoreceptor atrophy, which might facilitate the formation of a hole along with vitreoretinal interface abnormalities [13]. This was also speculated in other inherited retinal diseases, such as Best disease, in which MH is reported to be a rare complication​​​​​​​ [8,13].

## Conclusions

Inherited retinal dystrophies may be associated with MH formation, which may also be complicated by retinal detachment. Stable visual acuity following surgical intervention is possible without the worsening of the preexisting condition. Further studies, however, are prudent in understanding the pathophysiology of MH formation to prevent possible complications such as retinal detachment, especially in patients with multiple gene variants.
